# Supporting families and building relationships: Evaluation of a home and sleep safety equipment scheme for impoverished communities

**DOI:** 10.1016/j.puhip.2026.100733

**Published:** 2026-01-14

**Authors:** J. Bates, O.C. Kokogho, K.E. Dunstan-Smith, L. Davison, H.L. Ball

**Affiliations:** a Public Health Team, Durham County Council, UK; b Durham Infancy & Sleep Centre, Department of Anthropology, Durham University, UK; c Research & Public Health Intelligence Team, Durham County Council, UK; d Social Inclusion Team, Durham County Council, UK

**Keywords:** Child poverty, Sleep safety, Home safety, Infant mortality, Health inequality

## Abstract

**Objectives:**

Sleep safety and home safety resources allow families to care for babies and young children, preventing injuries and child death, adverse outcomes that are strongly linked to poverty and social deprivation. Parenthood involves unexpected costs and greater levels of unmet need for safety resources occur in low-income families. We evaluate a local authority scheme which enabled professionals in County Durham to apply for necessary safety equipment on behalf of eligible families.

**Study design:**

A holistic review of the first year of the operation of the Start For Life Fund (SFLF) scheme.

**Methods:**

A mixed method approach was used comprising: 1) a descriptive analysis of the application data submitted by professionals; 2) an online survey to capture the views and experiences of staff who had and had not used the scheme; 3) semi structured interviews with staff applicants and recipient families.

**Results:**

679 families (988 children) were supported during the first operational year, average cost £407 per family (£280/child). Three-quarters of children (72.3 %) were under three; over a third (35.7 %) were pre-birth to 1-year. Staff from seven services and over 20 job roles made applications for families with financial, relationship, housing, domestic violence, and disability-related needs, most from areas with high deprivation scores. 256 staff across 8 service areas submitted survey responses, 39 % of whom had used the scheme which was viewed extremely positively. Interviews with 13 staff and 7 families evidenced how children, families and practitioners benefitted. Recipients reported reduced stress and anxiety about child safety and increased parental confidence.

**Conclusions:**

By providing families with the sleep and home safety equipment they can't afford the SFLF gives parents the opportunity to change behaviours and reduce the risk to babies and children from unintentional injury and death. It helps to improve working relationships between practitioners and families, reduces parental experiences of anxiety, and risk to staff of moral injury. Taking steps to reduce unexpected infant death and child unintentional injury is crucial for families in absolute and relative poverty. Other local authorities could emulate this scheme.

**What this study adds**

• Evaluates a novel intervention to address infant mortality and child injuries at home using the principles of COM-B
• Identifies need for sleep safety and home safety equipment provision in areas of socioeconomic deprivation
• Expands understanding of the effects of poverty on children aged 0–5 years, and reduces exposure of staff to moral injury

**Implications for policy & practice**

• Infant deaths and child unintentional injuries need targeted prevention where families are living in absolute and relative poverty. Practitioners can identify and nominate at-risk families for rapid provision of safety provision
• Partnership working between Local Authority Social Inclusion Team and VCS equipment suppliers is effective
• Provision of infant/child safety equipment allows families to engage with practitioner guidance and helps to improve working relationships

## 1 Introduction

Information promoting infant sleep safety and child home safety frequently emphasises the importance of parental awareness, education, and responsibility [1,2]. The COM-B model identifies that behaviour is the interplay of capability, opportunity, and motivation [3], and in some cases, parents' opportunities to implement safety guidelines are limited, despite parental capability, and motivation to implement health-promoting behaviour. This may arise when financial limitations prevent parents from obtaining recommended safety equipment [4]. Such situations are also challenging for practitioners who may identify hazards in the child's home environment that parents are unable to resolve, leading to moral distress and injury for staff [5], and hindering positive practitioner-parent relationships.

Recent estimates suggest that around 25 % of the adult population has less than £100 in savings [6,7], and in the North of England children are more likely to live in poverty than the rest of the UK [8]. Birth and parenthood bring costs which can be unexpected and difficult to afford. The assistance available through the Sure Start maternity grant, child benefit and Healthy Start Voucher scheme do not fully cover the costs of parenthood for low-income families. The rates of both Sudden Unexpected Death in Infancy, and child in-home injury are strongly linked to poverty and social deprivation [9,10]. In County Durham in 2023-24, 27.1 % of children aged 0–4 years lived in absolute poverty and 30.7 % in relative poverty. The equivalent figures for England are 22.1 % (relative poverty) and 19.1 % (absolute poverty) [11].

**Fig. 1:**
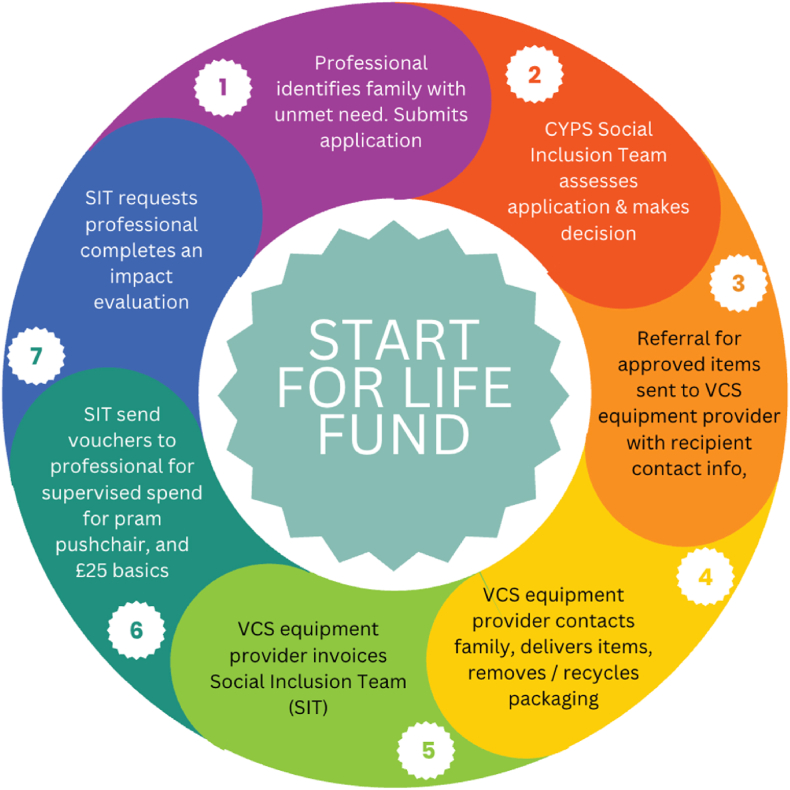
Start for Life Fund Scheme Process Diagram illustrating how DCC Children and Young Peoples Services Social Inclusion Team work with VCS furniture providers to deliver the scheme.

Research investigating how to reduce infant deaths and child accidents in the home environment among vulnerable families in the UK has primarily focussed on the provision of information to motivate parents to adopt recommended practices [[12], [13], [14], [15], [16]]. In the US and NZ several interventions have recognised financial need to be a barrier to reducing infant and child deaths and injuries, with studies demonstrating some success for programmes providing equipment such as safe sleep spaces [17,18]. Durham County Council Public Health team identified an unmet need for safety resources in families, and a potential for moral distress or injury to staff who were aware of risks to babies and children that they were unable to resolve. In response Durham County Council (DCC) working with Voluntary & Community Service (VCS) partners implemented the Start for Life Fund (SFLF) scheme--an evidence-based initiative to improve infant sleep safety and child home safety where parents cannot provide the necessary safety equipment. The scheme supports and promotes the UK safer sleep guidance [19], and the home safety equipment guidance [16]. Professionals working with families in County Durham submit online applications for families who meet the eligibility criteria, from the population of children aged 0–5 years estimated to be 29, 400 (Fig. 1) [20].

Information obtained from UK Public Health Leads via the Faculty of Public Health indicated that while some UK local authorities promote or collaborate with charity schemes that provide infant sleep safety and/or child home safety to families in need (personal communication), only one other UK LA Public Health run scheme was in operation (Blackburn with Darwen). To date no research has examined how staff and families use and respond to such local authority-led schemes, and whether parental opportunities to improve infant and child safety are realised via the provision of appropriate equipment. This article addresses this gap by reporting on an evaluation of Durham County Council's Start for Life Fund scheme to provide families with equipment to promote infant sleep safety and home safety. In accordance with the COM-B model the evaluation aimed to assess the ability of the scheme to provide parents with the opportunity they needed to implement infant sleep and child home safety practices, thus addressing an important child health inequality.

## 2 Methods

An independent evaluation of the DCC SFLF scheme was undertaken by Durham University's Infancy & Sleep Centre (DISC) between October 2024 and March 2025. Durham University and Durham County Council Research Ethics Committees provided ethical approval. An Information Sharing Agreement was established between Durham County Council and Durham University in line with best practice following the North-East sharing protocol, with support from the DCC Information Governance Team.

Application data, collated by the DCC Children and Young Peoples Services, Social Inclusion Team who manage the scheme, was anonymised by a DCC Public Health Intelligence Specialist to remove all personal data and standardise data fields. Information on MSOA deprivation was provided to the DISC team by the DCC Research and Intelligence team who follow a systematic process using the England indices of deprivation LSOA scores to assign an ID2019 score and rank to each local MSOA. There are 65 MSOAs in County Durham and MSOAs typically have a population of around 8000. The DCC Research and Intelligence team also provided ONS mid-2022 population estimates for the 0–5 population by MSOA, which was used as denominator data to calculate the application rate by MSOA. The DISC team conducted analyses of the scheme outcomes based on the data provided.

A Qualtrics™ survey was created to solicit views and experiences of staff eligible to use the scheme who had, and had not, made applications on behalf of families [21]. The survey aimed to a) capture staff views of the scheme and application process, b) obtain staff accounts of how families had benefitted, and c) explore which staff had not yet engaged with the scheme and why. Key domains involved awareness of the scheme and its purpose, knowledge about who was eligible and how applications could be made, experience of the application and assessment process, and inferred and observed benefits to recipients. The survey was piloted by DCC staff familiar with the SFLF scheme and DISC staff unfamiliar with the scheme. The survey was then distributed via email to all DCC and partner organisation staff working with families with follow up reminders 2 weeks later.

After the survey a semi-structured interview schedule was developed to gain a more detailed perspective. We used the quantitative survey data, plus free-text comments provided by survey respondents, to develop questions for the semi-structured interviews. These involved probing for explanations, opinions and experiences related to implementing the SFLF scheme (staff interviews) and receiving equipment (family interviews). The interviews were therefore used to complement the survey and provide greater depth. Interviews were conducted with a diverse range of self-selected participants including practitioners who had and had not utilised the scheme; families who had directly benefited from the fund; administrators and managers responsible for running the scheme; and VCS equipment suppliers contributing to the purchase and delivery of the items offered by the scheme. All practitioners, equipment suppliers and administrative staff were invited to take part via direct email invitations distributed by DCC; participants who volunteered to be interviewed received a Participant Information Sheet (PIS) via email. To recruit families, practitioner teams received a direct email from DCC asking them to nominate families who were recipients of the scheme and willing to participate in an interview. Families who volunteered received a PIS, a link to a form for capturing contact details, and received a £20 gift voucher on completion of the interview.

All staff interviews were conducted via Microsoft Teams by one DISC researcher for consistency. Staff interviews primarily involved probing for explanations, opinions and experiences related to implementing the SFLF scheme. Interviews with families were conducted by the same researcher over the phone, exploring parents’ experiences of receiving equipment provided by the scheme, and probing for descriptions of how infant sleep or child home safety issues were experienced before and after equipment provision. A semi-structured approach was used for all interviews, and informed consent was obtained before commencing. The discussions were audio-recorded with permission, and auto-transcribed. An experiential thematic approach was used to analyse the responses^22^ identifying experiences and insights from the topics discussed in the interviews using a realist perspective. Specifically, this involved the original interviewer coding each transcript iteratively to produce a set of codes reflecting the topics mentioned by the interviewees as examples of their individual experiences. These codes were verified by a second coder who independently coded a sub-set of the transcripts. Through a discursive process the two coders grouped codes into experiential themes relating to staff and to families.^23^

## 3 Results

Launched in September 2023, the SFLF supported 679 families (988 children) during its first operational year, with an average total cost of £407 per family (£280/child). One third of parent recipients were aged 18–25 (32.4 %), two-thirds were over 26 (62.4 %), with a small proportion under 18 (2.9 %). Over a third (35.7 %) of children supported were pre-birth to 1-year, with almost three-quarters (72.3 %) under three.

Applications were submitted by a wide range of professionals (Table 1). The most frequent reasons for families needing support were financial: receipt of an unexpected bill, unmanageable levels of debt, and job loss. Relationship breakdown, unexpected house moves, fleeing domestic violence, and illness or disability were also recorded.

**Table 1:** Roles of staff submitting SFLF applications on behalf of families between September 2023 and August 2024.

Role	n	%
Health Visitor	141	21 %
Social Worker	95	14 %
Key Worker	49	7 %
Nursery Manager	33	5 %
Family Support Worker	32	5 %
Midwife	31	5 %
Early Help Practitioner	27	4 %
Portage Education Worker	22	3 %
Family Health Nurse	20	3 %
Family Worker	20	3 %
Nursery Officer	20	3 %
Family Health Practitioner	19	3 %
Project Worker	12	2 %
Community Anchor	8	1 %
Maternity Care Assistant	8	1 %
School Project Manager	8	1 %
Community Group Advocate	8	1 %
Pastoral Lead	7	1 %
Support Co-ordinator	7	1 %
Treasurer	7	1 %
Financial Inclusion Advisor	6	1 %
Learning Mentor	6	1 %
Young Person's Advisor	5	1 %
Other	41	6 %
**Total**	679	100 %

Professionals assessed family equipment needs for both sleep safety (cots, Moses baskets, prams, beds, bed-guards, mattresses and bedding) and home safety (corner guards, door closure guards, socket covers, safety gates, fireguards, and blind cleats). Bedding, mattresses, and safety gates were the most frequently supplied items.

**Fig. 2:**
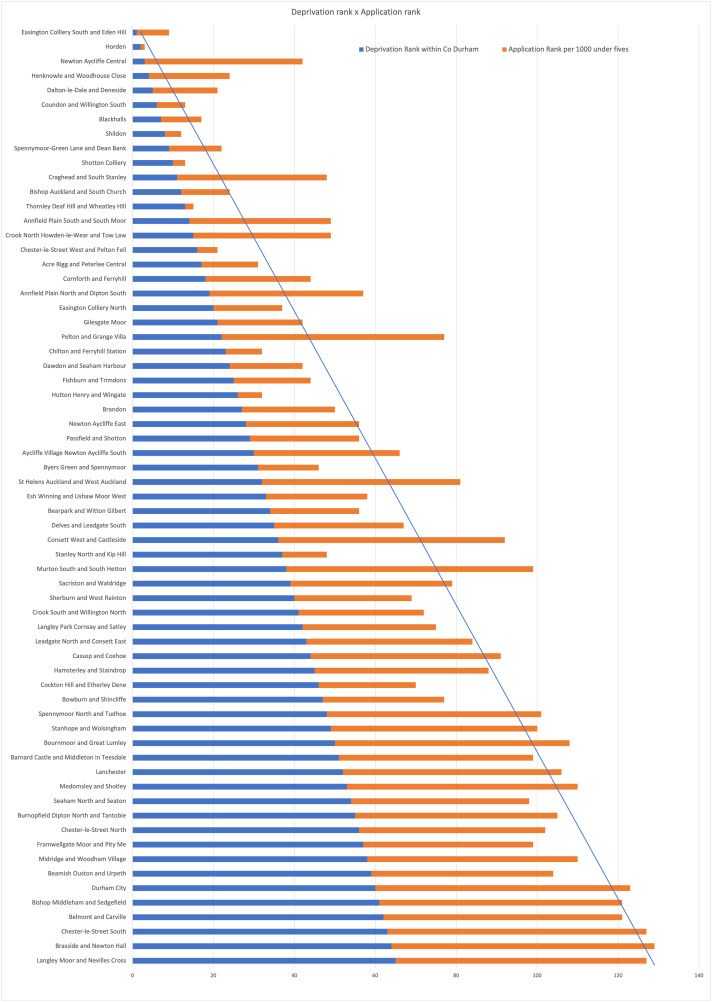
MSOA IMD rank against applications per 1000 children 0–4 rank.

The geographic distribution of applications was assessed for whether the scheme was reaching families in need across the whole county. Highest application rates mapped closely onto areas with greatest Index of Multiple Deprivation (IMD) scores. By plotting the IMD rank against application rank calculated per 1000 children 0–5 years in each MSOA we identified whether locations generated more or fewer applications than might be expected (Fig. 2). Apart from a handful of outliers the rankings for deprivation and applications were consistent.

### 3.1 Staff survey

256 valid survey responses were received from staff working in 8 broad service areas, with Children's Services staff comprising the largest group; 58 % of respondents were familiar with the scheme, 30 % were not, 12 % did not answer. Identical proportions of respondents knew where to find information about the scheme. Most staff were familiar with the scheme from DCC emails or from colleagues, but for 18 respondents the survey provided their first awareness. 39 % (100/256) of staff respondents had used the scheme while 61 % had not. Job roles of applicants and non-applicants are shown in Table 2.

**Table 2:** Job roles of survey respondents who had and had not applied to the SFLF scheme.

Job service area	Applicants n (%) n = 100	Non-Applicants n (%) n = 156	Total n (%) n = 256
Children's Services	52 (52 %)	77 (49 %)	129 (50 %)
Education Services	10 (10 %)	33 (21 %)	43 (17 %)
Health Visiting (0–19 Service)	16 (16 %)	2 (1 %)	18 (7 %)
Housing Services	4 (4 %)	1 (1 %)	5 (2 %)
Maternity Services	4 (4 %)	10 (6 %)	14 (5 %)
Mental Health Services	4 (4 %)	1 (1 %)	5 (2 %)
Other Services	0 (0 %)	15 (10 %)	15 (6 %)
Voluntary & Community Services	8 (8 %)	9 (6 %)	17 (7 %)
Blank response	10 (4 %)	2 (2 %)	8 (5 %)

Two fifths of applicants had submitted more than four applications each, while the majority had submitted between one and three. In 90 % of cases staff reported families were willing to have the application made, but some families (6 %) did not understand the scheme while the rest (4 %) needed different support. Staff who had submitted applications reported that the process was quick and easy to complete, and the guidance was easy to understand, while the number of challenges experienced was small. Staff felt that applications were processed quickly, and items were delivered to families promptly. Staff also reported that families had overwhelmingly positive experiences with the scheme: 93 % were fully or mostly satisfied, 94 % received equipment that met their needs, and 96 % felt the scheme had a positive impact on families.

Free-text comments at the end of the survey reflected high praise for the scheme, that it was highly needed, and an important resource for families with low incomes to make home environments safer for babies and young children in County Durham. Improving awareness across non-DCC staff groups was the biggest challenge to be resolved.

### 3.2 Staff interviews

Interviews were conducted with 11 practitioners who had used the scheme, 2 who had experienced similar schemes, and 7 families. Practitioners were very positive about the scheme (Theme 1). They consistently praised its ease of use, simple application, rapid turnaround, and the high quality of equipment provided. The scheme was viewed as both operationally efficient and highly valued by its users, reinforcing a strong desire for the scheme to continue. Benefits of the scheme for children (Theme 2), for families (Theme 3), and for themselves (Theme 4) were highlighted. Example quotes illustrating these themes are shown in Table 3.

**Table 3:** Participant quotes illustrating themes emerging from practitioner and family interviews.

Theme	Descriptor	Indicative quotes
Practitioner Theme 1	Scheme was highly valued	“The area we're working in … there's a lot of social deprivation and unemployment, and we have a lot of out-of-area families coming in who are maybe starting a new life so they haven't got a lot. So really for our area, definitely, everybody's been really pleased with it.” “I think the Start for Life Fund has been a fantastic fund. The fund is being really, really important. I think it's been great. Very positive.” “We all speak really positively about using it. “
Practitioner Theme 2	Scheme enhanced infant and child safety	“I have a little person who managed to turn a cooker knob on and set fire to a pizza box on the top of the oven before the safety gate. With the safety gate he can't get into the kitchen now. So yeah, so it's quite, it's quite big that one.” “One [case] was where the mum had the baby--she was only six months old at the time--she had her in the next- to-me, where they sleep beside you in a little separate thing on the bed. And the baby was ready to go into a cot because she was pulling herself up and kind of like was falling out. We requested the cot for her, which then meant she … wasn't able to climb out. The mum was thankful for that. So it reduced the risk of them falling out.”
Practitioner Theme 3	Scheme reduced financial burdens	“We work with a lot of under-5s and we're in an area that is faced with a lot of deprivation …. We go into a lot of houses that aren't necessarily suitable when it comes to safety … The parents … simply don't have the funding to go out and get those resources.” “They don't have the funds to buy these things themselves, where the little money that they do have is prioritized on food and usually using public transport to get to appointments, and heating, and those other things come kind of very low down on the list when you don't have much money.”
Practitioner Theme 4	Scheme improved practitioner-family relationships	“It definitely has given our practitioners the confidence to be able to have those conversations with families really, really, honestly. And then if they come across an issue, they're able to offer a solution to that issue. You can see that there's a need there.” “It really does help with the relationship with the practitioner and that then means that they're more open to be able to listen and take feedback on what's needed for the for the welfare of that child and for the for the welfare of the whole family. Helps build that empathy and that relationship with families.“
Family Theme 1	Scheme enhanced child safety and reduced parental anxiety	“I didn't realise they were going to bring like the corner covers and the TV strapping all that stuff. I thought it was just the baby gates being delivered, but I got more than I bargained for, to be honest, I got a door stopper that's on top of my living room door. That stops him from like catching his fingers in the door.”
Family Theme 2	Scheme supported parents by reducing financial burdens	“My son's bed broke. So I was saving up for another bed for him. You know, it was taking me time, and I definitely needed help.”
Family Theme 3	Scheme promoted parental confidence, pride, and positivity.	“The items I got were so overwhelming 'cause, you know, I didn't expect them to be like new. They were brand new items. When you hear about, you know, like schemes and charity, you think you know, it could be, like, second-hand or things like that. So, you know, it was brand new!”

Interviewees evidenced the scheme's role in enhanced child safety by reducing exposure to hazards such as burns and falls and by creating secure sleeping spaces for babies (Theme 2). One interviewee discovered a family had a cot with a bolt missing. The family had used string to hold the cot together, but the repair was unsafe, so a new cot was provided. Another family's toddler had learned how to turn on the hob burners, setting fire to some pizza boxes, so a safety gate was provided to prevent him accessing the kitchen. Administrator interviews confirmed that in a handful of cases very urgent applications had been actioned at speed with the support of the VCS equipment suppliers.

The impact of the scheme in supporting families with financial burdens was a widely mentioned benefit (Theme 3). Financial constraints in some households meant that items like fireguards, or safety gates were far down the list of necessities to be purchased. The scheme allowed families the opportunity to change behaviours (as per the COM-B model) on receipt of equipment from the scheme, as a bed for their child meant they could establish bedtime routines or reduce unsafe sleeping. Practitioners noted that the scheme helped families by reducing worries and building confidence and enabled families to be better able to work with practitioners.

In relation to household debt, scheme administrators noted that any application to the SFLF scheme indicating debt or bills as a financial reason for needing support was an opportunity to offer debt advice and assistance. To facilitate this an email was sent to the practitioner who made the referral, encouraging them to discuss these options with the family, and the national Healthy Start Voucher scheme is promoted to professionals as part of wider work to address child poverty.

Some practitioners interviewed identified that the scheme also benefitted themselves (Theme 4) by making it easier to have challenging conversations with families about infant and child safety, especially where financial constraints prevented families from purchasing safety equipment. As the scheme allowed practitioners to assist families in obtaining necessary equipment this strengthened their relationships with families; after receiving support through the scheme parents were more open to listening, accepting feedback, and engaging in discussions about their children's well-being.

### 3.3 Family interviews

Recruiting recipient families was challenging as many are unfamiliar with research, and wary of sharing personal information. Interviews continued until the volunteer pool was exhausted. Family interviews illustrated how the scheme transformed home environments and supported family well-being under three themes. Families experienced enhanced child safety and independence by receiving high-quality equipment that provided parents with the opportunity to implement safe sleeping and mitigate household hazards, also reducing parents’ stress and anxiety (which reflected their motivation but lack of opportunity to make these changes without support) (Theme 1). The scheme provided significant financial relief and contributed to home stability, enabling families to access essential items without the burden of excessive costs (Theme 2). Furthermore, the scheme fostered the establishment of healthy family routines and built parental confidence and pride by ensuring that children had safe, independent spaces and that families could create a nurturing home environment (Theme 3). Example quotes illustrating these themes are shown in Table 3.

## 4 Discussion

Staff using the SFLF scheme to support the families they worked with were exceedingly positive about its purpose and impact, as well as the ease of making applications, the quality of the equipment provided, and that this met families’ needs. Some staff were unaware of the scheme having missed communications and felt the scheme could be more prominently advertised within their networks. While the application process was considered easy, practitioners felt the follow up process, reflecting the need for the operations team to maintain an audit trail, was more onerous.

Interviews reinforced that the SFLF scheme was well regarded by practitioners and families alike. The sleep equipment ensured infants and toddlers had a safe sleep environment, allowing capable and motivated parents the opportunity to implement sleep safety and bed-time routines (COM-B). Home safety equipment prevented children from the possibility of experiencing burns, falls and scalds. The scheme was credited for improved working relationships between practitioners and families as the latter felt supported and the former could work more effectively when families had necessary equipment for their children. Families felt grateful for the support they receive from the scheme and saw the benefit of the items they received.

Application rates were closely correlated with areas of deprivation; we identified that some geographic areas with high numbers of applicants had active community organisations or DCC-led multiagency community development partnerships who could actively identify families eligible for the scheme. Working closely with community organisations and partnerships in areas where fewer applications were being submitted would be an avenue for ensuring more eligible families are supported.

The role of the SFLF scheme in appearing to strengthen relationships between families and practitioners reflects the COM-B model of behaviour change. To effectively engage with sleep safety and home safety recommendations families need to have the capability, the opportunity, and the motivation. Amongst families lacking financial resources, opportunity was a barrier to engagement as they could not purchase the equipment needed to practice safe sleep or prevent accidents. Via the SFLF scheme this barrier was alleviated, and practitioners reported that families were now motivated to work on implementing their other recommendations, which fostered positive and supportive relationships. This reflects the COM-B concepts of ‘environmental restructuring’ and ‘enablement’ [3], and supports NICE guidance on child home safety. Safe sleep equipment interventions in the US such as ‘Cribs for Kids’ have also produced positive results in fostering engagement with target populations experiencing financial hardship [17].

The intervention may also foster benefits beyond its specific safe sleep and home safety aims. Some qualitative data suggested that in providing a lower risk environment for their children the equipment alleviated anxiety about child safety in the home, with potential positive impact on parental mental wellbeing. Practical benefits may also accrue through being able to establish sleep routines, supporting parents in other areas of life, including child attendance at nursery and school. Such potential long-term outcomes of the scheme should be assessed in future. Following receipt of the formal evaluation report by DU the scheme was allocated core funding in the Public Health budget to ensure its sustainability. In addition to funding for provision of equipment, sustainability requires structure (leadership/organisational support & delivery mechanism) and process (clear process for staff and families) which have been strongly established for this scheme. In terms of scalability the intervention is delivered in a local authority with a population of 29,400 aged 0–5 yrs; the population, number of families supported and average cost provides sufficient information to guide other LAs considering implementing such an intervention.

### 4.1 Limitations

This study reflects the limitations of a post-hoc pragmatic evaluation. Although a controlled-comparative implementation approach would have allowed for a more rigorous evaluation, this was not feasible given the immediate need by DCC to address inequalities in infant and child safety. Therefore, findings are observational and experiential, and outcomes are indicative rather than conclusive. We acknowledge that as participation of staff and parents was voluntary, we could only capture the experiences of twenty individuals who were willing to be interviewed which may present a biased view. Furthermore, conducting research in impoverished communities is rife with challenges, including a reluctance by community members to divulge details of their lives to ‘outsiders’ or ‘the authorities’; fear and distrust are substantial barriers to research in the settings where families need support from this scheme, and so recruiting sufficient interviewees to reach data saturation, or obtaining a representative sample with the funding available for the evaluation was not possible. Future research may further explore and theorise the role of community partnerships and practitioner engagement in free equipment schemes and examine cost effectiveness of the intervention once data on unintentional injury rates and unexpected infant deaths are released for the relevant time periods.

### 4.2 Conclusions

The County Durham Start for Life Fund scheme provides a model that other local authorities with substantial child poverty and health inequalities could implement; ‘in-practice’ learning from this evaluation highlights that partnership with local third sector organisations facilitated timely equipment provision; that the provision of new equipment made parents feel they and their children were valued; and that professionals reported they were better able to work with families when they could suggest solutions to the problems they identified. Challenges involved the need to keep an audit trail, especially around supervised spend of vouchers for non-furniture items, which some staff found difficult.

This work has enhanced local understanding of the impact of child poverty on the 0–5 age-group. Often child poverty is depicted in terms of free school meals and lack of school shoes – however this evaluation highlights that ensuring parents can meet the safer sleeping and home safety needs of their children is crucial for families living in absolute and relative poverty who are unable to purchase the equipment necessary. The evaluation provides evidence of parents implementing injury prevention measures and guidance to reduce the risk of unexpected infant deaths in County Durham. Although this evaluation was unable to capture the experiences of all recipient families, the Start for Life Fund scheme has now been allocated core funding and is helping to reduce inequalities in sleep safety and home safety by providing equity of access to necessary equipment. Longer term evaluation of child injury and death rates will further enhance understanding of child poverty in the 0–5 age group.

## Ethical statement

Ethical approval was received for the evaluation of the DCC Start for Life Fund scheme by DCC and Durham University research ethics committees. Data sharing agreement and Data Management Plan were both agreed.

## Contribution statement

JB and LD designed the SFLF scheme. HB, JB, OK & KDS designed the evaluation. KDS extracted and cleaned the application data. HB analysed the application data. OK conducted the evaluation survey and interviews. HB analysed the survey data and OK analysed the interview data. HB, JB & KDS supervised the evaluation. OK, JB & HB wrote the evaluation report. HB & JB drafted the manuscript. OK, LD, KDS edited and approved the manuscript.

## Funding

Funding for the evaluation was provided to Durham University from the DCC public health budget.

## Declaration of competing interest

The authors declare that they have no known competing financial interests or personal relationships that could have appeared to influence the work reported in this paper.
